# Children with Cerebral Palsy can imagine actions like their normally developed peers

**DOI:** 10.3389/fneur.2022.951152

**Published:** 2022-09-06

**Authors:** Jessica Galli, Gioacchino Garofalo, Sara Brunetti, Erika Loi, Michela Portesi, Giovanni Pelizzari, Andrea Rossi, Elisa Fazzi, Giovanni Buccino

**Affiliations:** ^1^Department of Clinical and Experimental Sciences, University of Brescia, Brescia, Italy; ^2^Unit of Child Neurology and Psychiatry, ASST Spedali Civili of Brescia, Brescia, Italy; ^3^Division of Neuroscience, Università Vita-Salute San Raffaele, Milano, Italy; ^4^IRCCS San Raffaele, Milano, Italy; ^5^Department of Molecular and Translational Medicine, University of Brescia, Brescia, Italy

**Keywords:** motor imagery, Cerebral Palsy, action observation treatment, neurorehabilitation, action re-enactment

## Abstract

The present study aimed at assessing whether children with Cerebral Palsy (CP) can imagine object directed actions similarly to their normally developed peers. We asked children with CP (*n* = 12) and paired healthy controls (*n* = 12) to imagine in first person perspective eight daily actions, after observing them through videoclips presented on a computer screen. During motor imagery (MI) children were interrupted at a specific timepoint (e.g., at 2.5 s) from the start. Two frames extracted from the videoclips were then presented on the screen. One of the two depicted the correct timepoint at which the imagined action was interrupted, while the other represented an earlier or later timepoint. Children had to respond by pressing the key associated to the correct frame. Children also underwent VMIQ-2 questionnaire. Both groups performed similarly in the questionnaire and in the requested task, where they showed the same error rate. Errors mainly concerned the later frame, suggesting a similar strategy to solve the task in the two groups. The results support the view that children with CP can imagine actions similarly to their normally developed peers. This encourages the use of MI as a rehabilitative tool in children with motor impairment.

## Introduction

Motor imagery (MI) defines the capacity of an individual to “mentally rehearse simple or complex motor acts that are not accompanied by overt body movements” (1). It represents the voluntary effort of an individual to imagine himself/herself (1st person perspective) executing a specific action (2). MI should be disentangled from visual imagery that refers to the capacity to visually represent an action, by producing visual representation of the moving limb, in which case the individual is a spectator of the action (3rd person perspective). There is a general agreement that during MI, individuals recruit the same neural structures involved in the actual execution of the imagined actions [for pivotal studies see (3, 4); for review see (1, 5)]. A special point of interest is that, at least in adults, the time course of the imagined action follow that of the executed action [(6); for a review see (7)]. The same neural structures involved in the execution and imagination of actions are also recruited when other cognitive aspects of action, like action-observation and understanding, come into play and even when processing language describing those same actions [see for review (8–10)]. These neural structures involved in action execution as well as motor cognition (motor imagery, action observation and understanding as well as processing action related language) include frontal and parietal areas strictly interconnected (11).

The substantial motor equivalence between MI and action execution raises the issue whether patients with lesions affecting the neural structures normally involved in action execution can imagine actions or MI is equally impaired as the execution. In adults, pivotal studies (12–14) showed that a lesion in the neural circuits involved in action execution also affect MI. In keeping with these results obtained in adults, earlier studies concerning children with Cerebral Palsy (CP) showed that these children seem to have difficulties in imagining actions (15–17). However, more recent studies (18, 19) provided with different results, generally suggesting that children with CP can have a preserved capacity to imagine actions. More in details, the first study (18) stressed individual differences especially present in children with CP, when compared with normally developed children, thus highlighting that MI deficits are not universally observed in this population. The second study (19) showed that children with CP showed a general preserved capacity to imagine actions, although they committed more errors than normally developed children. The authors suggested that this can be related to differences in actual performance and working memory capacity between the two groups.

As a whole, it is still a matter of debate whether children with CP are able to imagine actions as their normally developed peers [see for review, (20)]. This raises the problem of using MI as a rehabilitative tool both in adults and children. In this respect there are several studies that have demonstrated the effectiveness of MI in the rehabilitation of different neurological diseases including stroke (21), Parkinson's disease (22), Multiple Sclerosis (23), and also in children with CP (24–26). However, in the light of the substantial overlap of the neural substrates subserving MI and actual action execution, the use of MI as a rehabilitation strategy for motor recovery has been questioned (27).

Given these contrasting results, the aim of the present study was to assess whether children with CP maintain the capacity to imagine actions and hence, at what extent, MI can be used for the recovery of motor impairment in childhood. For this purpose, we compared, in a case-control study, children with CP with their normally developed peers. At difference from previous studies, we assessed MI capacity by means a more ecological approach. To this aim, we used a novel task where we asked children in both groups to observe a goal directed action. We assessed their capacity to imagine themselves performing the seen action focusing, not only on the goal, but also on the temporal aspect (i.e., the time course) and duration of the action itself. Note that we did not ask for response times, but we only checked (see below Section Methods) for the correct mental execution of the task. Besides this novel approach that provided us a more objective index of the actual MI capacity by participants, we also collected the subjective description of children's capacity to imagine actions by means a well-established MI questionnaire.

## Methods

### Study design and ethics

A case-control study was conducted. Recruitment criteria and methodological procedures were approved by the Ethics Committee of the University Hospital of Brescia (Approval Number: 4014). The present study was conducted in accordance with the ethical standards of the institutional research committee and with the 1964 Helsinki Declaration and its later amendments.

### Participants

All children referred to the Unit of Child Neurology and Psychiatry at ASST Civil Hospital of Brescia with a diagnosis of CP from March 2020 to December 2021 were eligible. Inclusion criteria were the presence of CP confirmed by neuroimaging [computed tomography (CT) and/or magnetic resonance imaging (MRI)], Intelligence Quotient > 70, age between 7 and 12 years. Exclusion criteria were the presence of major visual and/or auditory deficits and drug treatment affecting the central nervous system. A total of 12 children (mean age 9.9 years, SD 1.67; 7 males, 5 females) met the inclusion/exclusion criteria and were enrolled. Five children had unilateral and 7 bilateral spastic CP. Three participants had left-sided hemiplegia, 2 right-sided hemiplegia, and 7 had diplegia. Full details of all enrolled children are shown in Table 1. Before entering the study, the parents of each child gave written informed consent. Twelve healthy children, matched by age, sex and school level, were also recruited as a control group (mean age 9.5 years, SD 1.62; 7 males, 5 females).

**Table 1:** Demographic data, clinical features, and radiological findings in participants.

**Patient no**.	**Sex (M/F)**	**GA (weeks)**	**Age (years, months)**	**CP type, Hagberg**	**Motor abnormalities: nature and typology**	**GMFCS/** **MACS/** **CFCS**	**Associated impairments**	**FIQ/ VIQ/ PIQ**	**Radiological findings (brain MRI)**
1	M	41.5	10.8	Left hemiplegia	Unilateral spastic hypertonia	1/2/1	V: CVI; H: no; LD: no; E: no	91/92/108	Right periventricular cystic leukomalacia, with triventricular hydrocephalus
2	F	33.5	7.9	Left Hemiplegia	Unilateral spastic hypertonia	1/2/1	V: no; H:no; LD: no; E: yes	93/110/76	Right ischemic frontoparietal malacic area with focal cortical atrophy, ipsilateral ventricular dilatation and right cerebral peduncle hypotrophy (Wallerian degeneration). Signal T2 and FLAIR hyperintensity in the right striatum and thalamus
3	F	33.5	11	Diplegia	Bilateral spastic hypertonia	2/1/1	V: CVI; H: no; LD: no; E: no	89/102/91	Periventricular leukomalacia, corpus callosum hypoplasia
4	M	39	7.2	Right hemiplegia	Unilateral spastic-dystonic hypertonia	1/2/1	V: CVI; H: no; LD: no; E: no	95/106/89	Internal capsule and corona radiata white matter involvement; left cerebral peduncle hypotrophy
5	M	32	12.8	Diplegia	Bilateral spastic hypertonia	1/1/1	V: no; H: no; LD: no; E: no	137/140/132	Periventricular leukomalacia, corpus callosum hypoplasia
6	F	27	11.3	Diplegia	Bilateral spastic hypertonia	2/1/1	V: yes; H: no LD: no, E: no	115/117/109	White matter hyperintensity of the temporal horn
7	M	31	10.7	Diplegia	Bilateral spastic hypertonia	3/2/1	V: CVI; H: no; LD: no; E: no	101/116/91	Periventricular leukomalacia, corpus callosum hypotrophy.
8	M	40	9.9	Left hemiplegia	Unilateral spastic hypertonia	1/1/1	V: no; H: no; LD: yes; E: yes	79/96/78	Right peri ventricular porencephaly; internal capsule and right cerebral peduncle hypotrophy (Wallerian degeneration).
9	M	29	9.3	Diplegia	Bilateral spastic-dystonic hypertonia	2/1/1	V: no; H: no; LD: no; E: no	121/114/129	Mild ventricular asymmetry (right > left)
10	M	34.4	7.6	Diplegia	Bilateral spastic hypertonia	1/2/1	V: yes; H: no; LD: no; E: no	133/148/122	Periventricular cystic leukomalacia
11	F	32	10.5	Right hemiplegia	Unilateral spastic hypertonia	1/2/1	V: CVI; H: no; LD: no; E: no.	84/96/89	Left putamen, corona radiata and nucleus caudate malacic areas with gliosis
12	F	41.4	10.11	Diplegia	Bilateral spastic hypertonia	2/1/2	V: no; H: no; LD; E: yes	Leiter-R 73	Right fronto-parietal, left occipito-parietal malacic area with gliosis, extended into the left caudate nucleus; enlargement of the left ventricle

M, male; F, female; GA, gestational age; CP, cerebral palsy; GMFCS, Gross Motor Function Classification System; MACS, Manual Ability Classification System; CFCS, Communication Function Classification System; V, vision; CVI, cerebral visual impairment; H, hearing; M/A, memory and attention; LD, learning disabilities (North American usage; mental retardation); E, epilepsy; FIQ, full Intelligence Quotient; VIQ, Verbal Intelligence Quotient; PIQ, Performance Intelligence Quotient; MRI, magnetic resonance imaging.

### Apparatus, stimuli, and procedure

The experiment took place in a dimly lighted room of the U.O. of Childhood and Adolescent Neuropsychiatry of Brescia, adequately prepared to make the children feel at ease. The room was free of elements that, possibly being in the field of vision of the child while he/she was observing the monitor, could distract her/him.

During the experiment, the child was sitting in front of a computer screen, on which the stimuli and the instructions were presented. Stimuli were short videoclips lasting 4 s, in which an actor performed common daily actions with different objects. Daily actions were chosen among those children are familiar with. Descriptions of the videoclips are reported in Table 2.

**Table 2:** List of actions presented through video clips and seen by children in the MI task.

**Action**
Grab and move a soft toy from right to left and vice versa
Open a jar
Take the chocolate bar and bring it to your mouth
Put on the line the LEGO-type bricks
Stack the LEGO-type bricks
Draw a semicircle on the sheet
Play with the ball by moving it from left to right and vice versa
Drive a toy car along the route

To provide their responses, children had to press one of two keys on the computer keyboard (“Q” and “Page Up”). The keys were colored in yellow and red, respectively. Keys were symmetrically placed with respect to the children's body midline.

**Figure 1:**
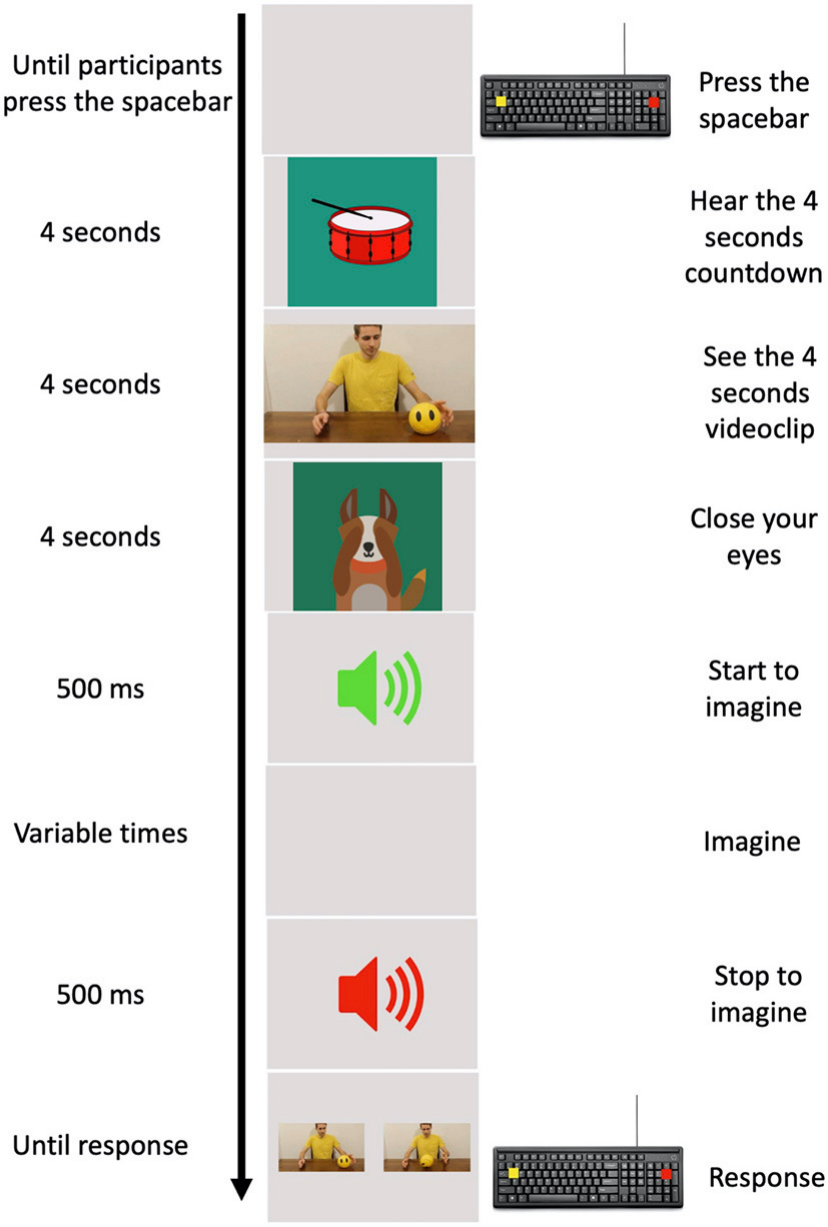
Experimental procedure. In the left column, the time of each event is reported. In the middle column, a pictorial example of the events is depicted. In the right column, the action requested by participants to provide their responses is reported.

The experimental task was implemented using PsychoPy 3.0 (28). A practice phase, in which children were trained to perform the task at their best, consisted in 10 practice trials that were not included in the experimental phase and were not analyzed further. An experimental trial started when the child pressed the spacebar. An animated percussion tambourine that rhythmically beat 4 shots (1 at s) appeared on the screen in order to give an auditory and mental timing and prepare the child for the clip. A 4-s clip sequence appeared on the screen where an actor performed one of the chosen daily actions. Following the clip, a cartoon of a little dog appeared on the screen which, bringing its paws to its eyes, invited the child to close his/her eyes, too. An auditory signal (Start signal) indicated to the child to start to imagine the previously seen action respecting the temporal features of it. It is worth stressing that, we required participants to imagine actions in first person perspective (i.e., participants had to imagine themselves performing the action). During motor imagery, a second auditory signal (different from the previous one, Stop signal) was presented at different randomized time intervals (1.5, 2, 2.5, 3, and 3.5 s). This second auditory signal indicated the moment in which children had to stop to imagine the seen action and re-open their eyes. Following the Stop signal, two pictures depicting two frames of the previously seen action appeared on the monitor. One of these corresponded to the exact moment in which the action was interrupted, the other depicted a moment 750 ms earlier or later than the exact one (see Figure 1).

The child was asked to choose the picture representing the exact moment when the action was interrupted by pressing one of two colored buttons on the keyboard. We also counterbalanced the position of the correct picture representing the exact moment in which the action was interrupted, presenting it on the screen either on the left or on the right. As a whole, children performed 160 trials obtained from the combination of the 8 actions, ×5 different randomized time intervals at which the action could be interrupted, ×2 frames that could be either earlier or later than the exact one, ×2 positions of the correct frame. In order to avoid possible mental fatigue, the task was set to allow children to have a rest whenever they wanted or needed at the end of each trial during the experiment.

All children enrolled also underwent the two scales of the Vividness of Movement Imagery Questionnaire-2 [VMIQ-2; (29)] aimed at assessing their self-reported capacity to imagine actions. In Scale 1 (EVI scale, External imagery) children are requested to imagine themselves performing an action from a third-person perspective; in Scale 2 (KIN scale, Kinesthetic imagery) they are requested to imagine themselves performing an action from a first-person perspective.

### Analyses

Data analysis has been performed using R 4.0.4. Children's error rate in the task was recorded and analyzed. We considered errors the choice of the frame that did not depict the exact moment in which the imagined action had been stopped. We excluded from the analysis practice trials. Two participants (one in the control group and one in CP group) have been excluded from analysis since they did not complete the task.

Given the design of the experiment (multiple observation for participants and stimuli) and the characteristic of the distribution of the errors (binomial distribution), we modeled the data using a multilevel logistic regression. The selection of the model that best expresses the plausibility of our data with respect to the variables considered was made taking into account the Bayesian index [BIC, (30)]. The choice to use the Bayesian model lies in the fact that this predicts with equal probability the a-priori likelihood of the null hypothesis (H0) and of the alternative hypothesis (H1).

The uncertainty of the model has been evaluated through Bayes weights, which can be considered analogous to an estimate of the probability that a given model is the best model that yields the data. Therefore, if a model is associated with a Bayes weight >0.95, it is considered the only valid data model. If no model reaches this criterion, all models are ranked from the best to the bottom, proceed along the list until the cumulative weight of Bayes exceeds 0.95 and the rest is rejected. This defines a “confidence set” of 95% models (31), meaning we can be 95% sure that one of the models in the set is the best approximation to the data.

The full model has been implemented with Group (with 2 levels: Cerebral Palsy vs. Control) as a between-participant factor, and Time-point (i.e., if the alternative response to the correct one was a frame depicting a time point that preceded or followed the correct one, 2 levels: earlier vs. later) and Stop Time (i.e., after how long the sound signal that interrupts the imagination is presented to the participant; 5 levels: 1.5, 2, 2.5, 3, and 3.5 s) as within-participant factors. Participants were set as random effect. Model selection was performed using dredge() function of MuMIn package (32).

We further investigated the capacity to imagine actions in both groups using the VMIQ-2. Questionnaire scores has been analyzed running a between groups *t*-tests (Control vs. CP) for both scales of the questionnaire.

## Results

**Figure 2:**
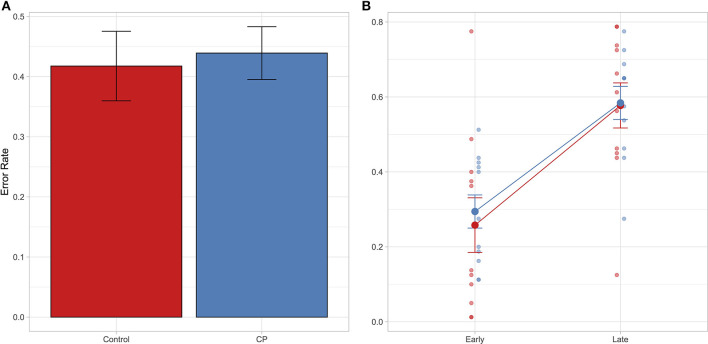
In both panels it is reported the Error rate index calculated as follows: Incorrect response/(Correct response + Incorrect response). In **(A)** mean Error rate is reported as a function of group. In **(B)** mean Error rate is reported as a function of time-point and group. Error bar referred to the standard error of means. Individual data are also shown.

Results showed that the best model that yields our data is the model including the factor Time-point (BIC = 4,534.1, Bayes weight = 1). Interestingly, no difference between group has emerged: both groups make the same rate of errors as shown in Figure 2A. Furthermore, this similar pattern is replicated even if we explore the interaction between Group and Time-point (see Figure 2B), confirming the result of the model selection.

Results of the VMIQ-2 did not show any significant differences between groups [Scale 1—EVI: *t*_(1, 19.91)_ = 0.14, *p* = 0.89; Scale 2—KIN: *t*_(1, 19.91)_ = 0.35, *p* = 0.73], confirming that both healthy children and children with CP can image the actions described in the questionnaire in both scales (EVI: M_cp_ = 3.90, SD = 0.46; M_control_ = 3.89, SE = 0.45; KIN: M_cp_ = 3.95, SD = 0.32; M_control_ = 4.01, SD = 0.34).

## Discussion

The present results support the notion that children with CP can imagine actions similarly to their typically developed peers. Overall, both groups showed no differences in the capacity to imagine actions as revealed by the VMIQ-2 questionnaire. Even more interesting is the evidence that children in the two groups obtained the same results in the novel MI task we delivered and shared the same strategy to solve it. In details, children with CP and their peers made the same number of errors. These mostly occurred when children had to judge the picture depicting the later frame. There were less errors when they had to judge the earlier frame. In other words, in both groups the timing of the imagined actions was faster than the timing of the seen action. These findings strongly suggest that, while imagining actions, both groups were strictly anchored to the goal of the action, so that they tended to anticipate the final part of it (i.e., hand-object interactions) and the imagined action resulted globally faster than the observed counterpart.

The present findings seem to be in contrast with those of previous studies showing that children with CP are not able to imagine like their peers. Following these studies, children with CP show the same behavior of adults with impairment of the motor system [see for example (12–14)].

Indeed, it is rather difficult to offer a clear-cut explanation for the present results. In our view, they may have two, not-mutually exclusive, explanations. The first one is related to the task required by participants. In previous studies, MI was assessed by means of rotational tasks and questionnaires [e.g., (15, 33)]. Rotational tasks seem to assess, not only MI, but also other cognitive domains as spatial perspective taking, visual imagery and the process of object-related features. However, using a rotational task, two very recent studies, reported results in keeping with the present ones (18, 19). It is worth noting that, these studies used response times to assess MI ability, while in our task we focused on the capacity of children to follow, during MI, the exact time course of the actions shown in the videoclip. This in the attempt to limit the influence of other cognitive processes potentially affecting the response times and error rate, as suggested by Souto et al. (19).

The second explanation could be related to differences in the way through which adults and children compensate motor impairment. In adults a lesion focusing on neural structures subserving motor functions can affect the capacity to imagine actions (12), to understand actions (14) and to process language related action (34). There is some evidence that this is not the case in children. For example, in an objective task involving imagination of walking in children with CP (35), authors did not find any difference in the duration of actual walking and imagined walking. This suggests that children with CP were able to use MI in an explicit task and that this kind of task may reveal the actual capacity to imagine actions.

In keeping with this, in children with CP, during a MI task in 1st person perspective, in an fMRI study it was found the activation of fronto-parietal areas known to be involved in action execution, with a slight left hemisphere prevalence (25). In a similar vein the same areas are shown to be involved also in action observation [(36), see also (37)]. Action observation treatment has been shown to be effective in the recovery of upper limb motor impairment in children with CP (38–40). Furthermore, following a rehabilitation training with action observation, treated children showed activation of fronto-parietal areas stronger than controls (41). It is worth stressing that all these findings point to a reenactment of fronto-parietal areas during motor imagery and action observation and understanding, thus supporting the notion that in children with CP a substantial overlap between areas normally involved in action execution and cognitive aspects of actions is still preserved, despite any potential impairment in motor execution. In a developmental perspective, these findings are in keeping with the results obtained in adults with congenitally absent or shortened upper limbs [e.g., (42)]. Even though, they had no (or very limited) capacity to execute upper limb actions, these individuals could understand and memorize upper limb actions as typically developed individuals do. The authors interpreted their results in favor of the notion that action understanding/processing is independent of action execution or motor experience, and, as a consequence, that the first task is disentangled from the second because it does not require the integrity of the neural structures sub-serving the second one (42).

The present findings obtained in children with CP and the results of studies in adults with congenitally absent or shortened upper limbs seem to suggest that when motor impairment (whatever the underlying disease or cause) occurs at an early stage in the development, individuals may preserve the capacity to build up an internal representation of actions based on the observation of actions performed by other people, by listening to the sounds of actions and finally by verbally describing them, in such a way that the motor equivalence between action execution and the processing of actions is preserved. In our view, all these data, rather than supporting the dissociation between action execution and motor imagery, action observation, action understanding and the understanding of action related language, should open the way to studies aimed at assessing the mechanisms of neural plasticity occurring at an early developmental stage.

The results of the present study are also relevant for the rehabilitation of children with CP for at least two reasons: first, by showing that children with CP are able to imagine actions in a manner similar to their healthy peers, these findings support the view that MI can be exploited as a rehabilitative strategy for the recovery of motor functions in these children; second, if one assumes that action observation and recognition, motor imagery and processing action related words share common neural mechanisms and possibly neural substrates (8, 9), then the present findings support the notion that the internal representation of actions is well preserved in children with CP, despite their impairment in motor execution. This evidence may further prompt the use of healthy models during action observation and motor imagery training, rather than models tailored to the kind of motor impairment evident during action execution (39, 43). Future studies should define how motor imagery and action observation can be used in a complementary way to provide the best practice for children or which subgroups of patients may better benefit from one or the other approach.

Despite the present findings seem to be relevant from a theoretical point of view, as well as for its clinical implications, some limitations should be underlined. Our sample was rather small and, indeed, future studies should aim at enlarging the number of children recruited and assessing the reliability of the task we proposed. Moreover, as for other studies of similar kind, our inclusion criteria were rather stringent and limited participation to children with CP who were not cognitively impaired. For this reason, the results of the study and the interpretation we forward of the findings can be generalized to children with CP who present with no or minor cognitive impairment and are potentially compliant with the requests of the task.

## Data availability statement

The raw data supporting the conclusions of this article will be made available by the authors, without undue reservation.

## Ethics statement

The studies involving human participants were reviewed and approved by Ethics Committee of the University Hospital of Brescia. Written informed consent to participate in this study was provided by the participants' legal guardian/next of kin.

## Author contributions

JG: project administration, resources, supervision, methodology, and writing—original draft. GG: data curation, formal analysis, methodology, visualization, software, and writing—original draft. SB, EL, GP, and AR: data curation, resources, and investigation. EF: project administration, supervision, writing—original draft, and writing—review and editing. GB: conceptualization, methodology, supervision, writing—original draft, and writing—review and editing. All authors contributed to the article and approved the submitted version.

## Conflict of interest

The authors declare that the research was conducted in the absence of any commercial or financial relationships that could be construed as a potential conflict of interest.

## Publisher's note

All claims expressed in this article are solely those of the authors and do not necessarily represent those of their affiliated organizations, or those of the publisher, the editors and the reviewers. Any product that may be evaluated in this article, or claim that may be made by its manufacturer, is not guaranteed or endorsed by the publisher.
